# Current and lasting effects of affect labeling on late positive potential (LPP) amplitudes elicited by negative events

**DOI:** 10.1002/brb3.3065

**Published:** 2023-05-14

**Authors:** Jiafeng Liang, Huiyan Lin

**Affiliations:** ^1^ Department of Applied Psychology, School of Education Guangdong University of Education Guangzhou China; ^2^ Institute of Applied Psychology, School of Public Administration Guangdong University of Finance Guangzhou China; ^3^ Laboratory for Behavioral and Regional Finance Guangdong University of Finance Guangzhou China

**Keywords:** affect labeling, current effects, lasting effects, LPP, negative pictures

## Abstract

**Introduction:**

Labeling the emotional aspect of self‐unrelated stimuli (i.e., affect labeling) is a crucial strategy for implicit emotion regulation. However, it is uncertain whether affect labeling influences event‐related potential (ERP) responses (e.g., the late positive potential, LPP) to negative stimuli in comparison with control conditions in which attention is shifted to the emotional content of the stimuli (e.g., affect matching). Additionally, it is unknown whether affect labeling has a lasting effect on the processing of negative stimuli.

**Methods:**

Participants were required to label the emotion (negative or neutral) of target pictures with two words, to match the emotion with alternative pictures or to merely view the target pictures. Target pictures were presented again immediately after the regulation task. After all the target pictures had been labeled, matched and viewed, the pictures were re‐exposed for the third time.

**Results:**

The results showed that negative pictures elicited larger late LPP responses during the affect labeling task than during other tasks. However, the LPP responses were smaller for negative pictures in the affect labeling condition than in the other conditions when target pictures were re‐exposed immediately after the task. When target pictures were re‐presented again long after the regulation tasks, the LPP responses were smaller for negative stimuli with a history of affect labeling than viewing, whereas this effect did not differ between the affect labeling and matching conditions.

**Conclusion:**

The current findings suggest that affect labeling has current effects and, to some extent, has lasting effects on negative stimulus processing.

## Graphical abstract

Negative pictures elicited larger late LPP responses during the affect labeling task. During the later re‐ exposure phase, the LPP responses were smaller for negative stimuli with a history of affect labeling. The current findings suggest that, to some extent, affect labeling has current and lasting effects on negative stimuli.

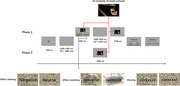

## 1 INTRODUCTION

Affect labeling, which contributes to managing unwanted emotions and the distresses associated with aversive events, is thought to be a critical strategy of implicit emotion regulation (e.g., Gyurak et al., 2011). Affect labeling can involve labeling one's own feelings and labeling the affective content of self‐unrelated stimuli (Torre & Lieberman, 2018). Several studies have investigated the former case of affect labeling (Burklund et al., 2014; Herbert et al., 2013; Memarian et al., 2017), but more interest has been paid to the latter case (e.g., Constantinou et al., 2013, 2014, 2015; Cuthbert et al., 2000; Hajcak et al., 2006; Herbert et al., 2013; Lieberman et al., 2007, 2011; Torrisi et al., 2013; Tupak et al., 2014). The present study mainly investigates the affect labeling of self‐unrelated stimuli.

Previous studies have investigated whether affect labeling influences event‐related potential (ERP) responses to self‐unrelated emotional stimuli. One critical emotion‐related component is late positive potential (LPP), a component that starts approximately 400 ms after stimulus onset and is distributed over parietal scalp sites. Previous studies have repeatedly revealed that the LPP response is stronger for emotional as compared to neutral stimuli, suggesting attentional engagement with salient environmental stimuli (Bradley, 2009; Codispoti et al., 2007; Foti et al., 2009; Schupp et al., 2000, 2004). Nevertheless, when the LPP was separated into different time windows (e.g., early, middle, and late), several studies showed that the emotional effect was evident only at an early LPP time window (< 1–1.5 s) but not at subsequent time windows (Cao et al., 2020; Ferri et al., 2012; Weinberg et al., 2012).

Moreover, this LPP component is also thought to be an index of emotion regulation (for reviews: Hajcak & Foti, 2020; Hajcak et al., 2010). It has been found that enlarging emotional responses to unpleasant stimuli enhanced LPP responses, whereas suppressing or decreasing emotional responses reduced LPP responses (Moser et al., 2006, 2009). These findings indicate that conscious upregulation increases emotional reactivity, while downregulation decreases the reactivity. Moreover, the effects of emotion regulation strategies were found to be different across the time windows of LPP (Paul et al., 2013; Schönfelder et al., 2014; Thiruchselvam et al., 2011). For example, distraction influenced the processing of unpleasant pictures in the early and middle time windows of LPP (approximately 300–4000 ms) but not in the later time window (> 4000 ms; Thiruchselvam et al., 2011). Reappraisal did not influence the processing of unpleasant pictures in an early time window of LPP but a later time window (Paul et al., 2013; Schönfelder et al., 2014; Thiruchselvam et al., 2011). Nevertheless, the timing effect on LPP responses was not observed when emotional stimuli were re‐exposed again (Thiruchselvam et al., 2011).

Using this index, Hajcak et al. (2006) showed a greater LPP response to both negative and positive pictures when labeling the emotional aspect of these pictures (i.e., the affect labeling condition) than when labeling the number of persons in the pictures (i.e., the control condition). This finding suggests that affect labeling enhances attentional processes for emotional pictures. In another study by Herbert et al. (2013), participants were presented with a face after being presented with a label regarding another person's emotion (e.g., his/her fear; the affect labeling condition [sender‐related labeling in the manuscript]) or after the letters “XXXX” (the control condition). With respect to the affect labeling condition, the participants were required either to view the labels and facial expressions (automatic labeling) or to label the emotion of the other person while looking at the faces (intentional labeling); in the control condition, they were only required to view the faces. In contrast to Hajcak et al.’s (2006) study, the results showed that LPP responses elicited by emotional faces were less positive in the automatic affect labeling condition than in the viewing condition, implying reduced attentional processes for emotional faces by automatic affect labeling. There were no differences between the intentional affect labeling and control conditions.

Therefore, the findings regarding the LPP effects of affect labeling remain inconsistent. The discrepant findings might be associated with whether affect labeling starts before or after the presentation of target stimuli [in addition to task demand (i.e., automatic and intentional affect labeling)]. In Herbert et al.’s (2013) study, the labels were preceded by target stimuli and the emotional content of the target stimuli was indicated by the labels. In this case, affect labeling might have taken effects prior to the presentation of the target stimuli (Lin et al., 2012, 2015, 2018, 2020). Due to the lack of prior labels in Hajcak et al.’s (2006) study, participants knew about the emotional content of target stimuli after their occurrence, thus, affect labeling only started after these occurrences. The different starting points of affect labeling might influence the processing of emotional stimuli and thus, LPP responses.

Notably, the LPP effect in previous studies essentially was considered in relation to differential responses between the affect labeling and control conditions (Hajcak et al., 2006; Herbert et al., 2013). Regarding the control condition, attention was shifted to the nonemotional content (i.e., the number of persons) of the target stimuli in Hajcak et al.’s (2006) study. In Herbert et al.’s (2013) study, attention focus was not required and thus, attention might be shifted to either the emotional (e.g., facial expression) or nonemotional contents (e.g., facial identity) of the target stimuli or both. However, affect labeling requires attentional focus on emotional contents. Using a control condition that requires direct attention to the emotional contents of target stimuli might provide a better understanding of the LPP effect of affect labeling.

More importantly, previous studies have indicated that some regulatory strategies have not only current but also lasting effects on emotional stimuli (e.g., Dunning & Hajcak, 2009; Foti & Hajcak, 2008; Moser et al., 2009; Paul et al., 2013; Schönfelder et al., 2014; Thiruchselvam et al., 2011). For example, distraction currently reduced LPP responses to negative stimuli during the regulation phase (Dunning & Hajcak, 2009; Paul et al., 2013; Schönfelder et al., 2014; Thiruchselvam et al., 2011). When the stimuli were presented again during the re‐exposure phase, however, LPP amplitudes were observed to be larger for negative pictures with a history of distraction than for those pictures in a history of viewing (Thiruchselvam et al., 2011). Nevertheless, it remains unknown whether there are any lasting effects of affect labeling on LPP amplitudes to emotional stimuli when they are re‐exposed subsequently.

Therefore, we aimed to investigate whether affect labeling has current and lasting effects on emotional stimuli compared to a control condition in which attention is directed toward the emotional content of the stimuli (e.g., affect matching). Affect matching refers to matching a target stimulus with another stimulus with reference to the emotional content. Similar to affect labeling, affect matching also requires attention toward emotion characteristics (Hariri et al., 2000, 2003; Lieberman et al., 2007; Torrisi et al., 2013; Tupak et al., 2014). Affect matching has been repeatedly used as a control condition for affect labeling in functional magnetic resonance imaging (fMRI) studies. Related studies have shown that affect labeling reduces the activation of amygdala and increases the activation of prefrontal cortex to a larger extent than affect matching, possibly suggesting reduced emotional responses as a results of increased inhibitory and executive processes (Hariri et al., 2000, 2003; Lieberman et al., 2007; Torrisi et al., 2013; Tupak et al., 2014). Viewing was also used as a control condition in the present study because this condition helps to determine whether the differences between the affect labeling and matching conditions are associated with affect labeling or matching (e.g., the differences would be associated with affect labeling if the viewing condition differs with the affect labeling but not matching condition, but with affect matching if the viewing condition differs with the affect matching but not labeling condition). Previous studies have also used the viewing condition as a control condition (e.g., Burklund et al., 2014; Lieberman et al., 2007, 2011).

To understand the current and lasting effects, participants in the present study were presented with negative or neutral target pictures on the upper part of the screen. Below the target pictures, each label (e.g., “negative” and “neutral”) was overlaid onto a scrambled picture in the affect labeling condition. In the affect matching condition, the abovementioned labels and scrambled pictures were replaced with the letter “X” repeated eight times (i.e., “XXXXXXXX”) and alternative pictures, respectively. For the viewing condition, eight letters were overlaid onto two scrambled pictures. The letters and scrambled pictures were used to reduce the differences in visual features among the conditions. After each target picture was labeled, matched or observed, participants were required to view the target picture again, but without any labels, letters or scrambled or alternative pictures, in order to understand how the effect of affect labeling was like shortly after the regulation task. After all target pictures had been labeled, matched or observed, participants were re‐exposed to the target pictures for the third time. This re‐exposure was used to investigate whether the effect of affect labeling could be sustained long after the regulation task.

For the current effect, because the present study did not present labels prior to emotional stimuli, we predicted that affect labeling would increase attentional processes over negative stimuli and thus LPP responses to a larger extent than the control conditions, in line with the study by Hajcak et al. (2006). Moreover, a model of affective adaptation suggests that emotional stimuli that have been previously attended to and whose emotional significance has already been evaluated lead to weaker emotional reactivity (Wilson & Gilbert, 2008). Thus, we predict that when the participants are re‐exposed to the related emotional stimuli after the regulation task is completed, preceding affect labeling might reduce emotional responses evoked by negative stimuli, and thus LPP responses.

## 2 METHODS

### 2.1 Participants

Thirty‐four healthy undergraduate students were recruited as participants and received course credits. Two participants were excluded due to excessive artifacts in the EEG signals (rejection rates > 30% of trials). Thus, the reported data were from 32 participants (18–23 years, *M* = 19.97, *SD* = 0.96; 20 females), for which power calculations using G*Power (version 3.1.7; Faul et al., 2007) showed a power of >85% to detect a small to medium effect size (*f* = 0.20) for the interaction between “experimental block” and “emotion category.” All participants were right‐handed with normal or corrected‐to‐normal vision. Participants did not report a history of neurological illness or mental disorders or were not taking medication. All participants provided written informed consent. The study was approved by the local ethics committee.

### 2.2 Stimuli

For the current study, stimuli contained 500 pictures. Four hundred pictures (200 negative and 200 neutral) were obtained from the International Affective Picture System (IAPS; Lang et al., 2008), and the other 100 were scrambled pictures. The IAPS pictures selected for this study contained animals, buildings, objects, persons, natural scenes, etc. Each picture had a width of 9.03 cm and a height of 6.77 cm (resolution = 28.35 pixels/cm).

Based on the normative valence ratings of the IAPS pictures, the negative pictures (*M* ± *SD* = 2.91 ± 0.77) were rated as more unpleasant than the neutral pictures (5.04 ± 0.29; *F*(1, 398) = 1362.05, *p* < 0.001,${\;\eta }_{p}^{2}$ = 0.77). The arousal ratings were higher for the negative pictures (5.66 ± 0.86) than for the neutral pictures (3.69 ± 0.92; *F*(1, 398) = 486.76, *p* < 0.001,${\;\eta }_{p}^{2}$ = 0.55).

The pictures were pseudorandomly separated into 4 sets with 50 negative and 50 neutral pictures each. For each emotional category of pictures, the valence and arousal ratings of the sets were not significantly different (*p*s ≥ .830). Three sets were used as target pictures and were distributed randomly to the affect labeling, affect matching or viewing conditions. The fourth set of pictures was used as alternative pictures in the affect matching condition during the regulation task.

In addition, we copied alternative pictures to create scrambled pictures using the Scramble Tool in Adobe Photoshop CS6. Alternative and scrambled pictures were then made translucent using Adobe Photoshop CS6 (the transparency was 60% of the original pictures) so that the participants could distinguish the (alternative and scrambled) pictures with labels/letters more easily.

### 2.3 Procedure

**FIGURE 1:**
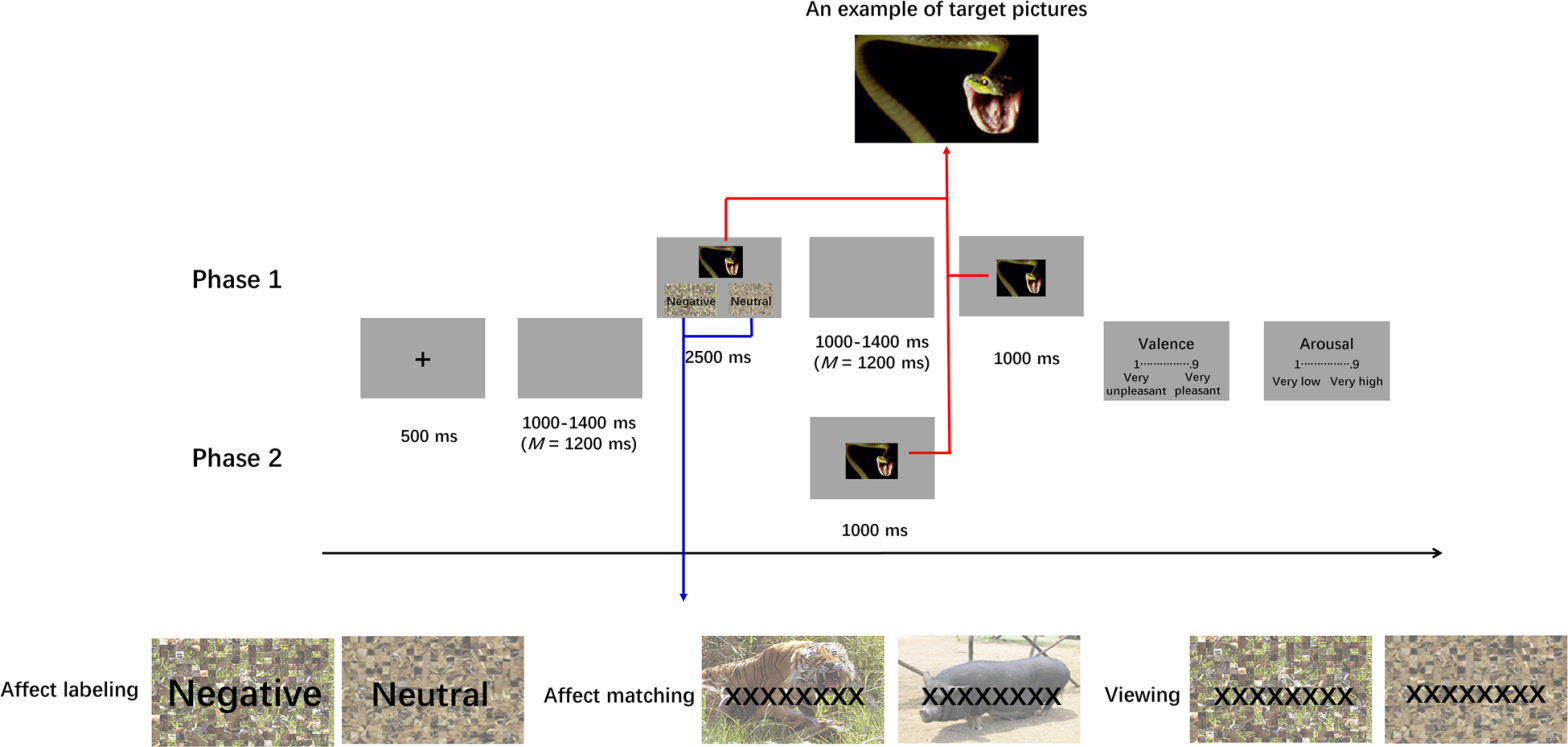
Experimental procedure of the regulation and re‐exposure phases.

As illustrated in Figure 1, the experiment contained Phase 1 and Phase 2. In Phase 1, there were three blocks of tasks (i.e., affect labeling, affect matching and viewing) whose presentation sequence was randomized across participants. Before the actual experiment for each block, the participants were informed about the instructions and performed 8 practice trials. Each trial started with a black cross for 500 ms. After a black screen was presented between 1000 and 1400 ms (*M* = 1200 ms), a negative or neutral target picture was presented in the upper portion of the screen for 2500 ms.

In the affect labeling block, the label “negative” or “neutral” was presented below the target picture on the left and right sides, and the presentation position (i.e., left or right side) of the labels was randomized. The participants were informed to choose a label to indicate the emotional content of the target picture by pressing the “F” or “D” key for the right or left label, respectively. In the affect matching block, two alternative pictures were presented below the target picture. One of the alternative pictures was negative, and the other was neutral. In half of the trials, negative alternative pictures were presented on the left side and neutral pictures were presented on the right side, while for the other half of the trials, the presentation position of the pictures was reversed. In this condition, the participants were asked to choose the alternative picture for which the emotional content was similar to the target picture by pressing the “F” or “D” key for the left or right alternative picture, respectively. In the viewing block, the participants were asked to view the target picture and to press the “F” or “D” key after viewing it. Participants could choose which key they press. The key pressing was to reduce additional differences (e.g., finger moving) between this condition and the affect labeling and matching conditions and to maintain participants’ attention to the stimuli. For the affect labeling and affect matching blocks, but not the viewing block, participants were required to respond as fast and accurately as possible.

After a blank screen was presented between 1000 and 1400 ms (*M* = 1200 ms), target pictures that had just been presented were presented again for 1000 ms, without any other stimuli. Notably, the presentation duration of target pictures was shorter in this presentation (and during Phase 2) than in the first presentation of target pictures. This was because participants were not asked to perform any tasks during this presentation of target pictures (and during Phase 2), but they were asked to perform tasks during the first presentation. Performing tasks required more time. The participants were then required to provide valence (“1” = “very unpleasant,” “9” = “very pleasant”) and arousal ratings (“1” = “very low,” “9” = “very high”) of the pictures on a 9‐point scale.

After Phase 1, the participants were informed about Phase 2. The participants were not informed about this task until this point in the experiment. They were told that the target pictures presented in the preceding phase would be presented again, but there would be no tasks (e.g., affect labeling, affect matching or viewing tasks) before or during the presentation of the pictures. Each trial was initiated with a fixation cross for 500 ms. Following a blank screen for 800–1200 ms (*M* = 1000 ms), a target picture was presented for 1000 ms. The sequence of target pictures in each block was randomized across participants. The participants were then required to provide emotional arousal and valence degree for the pictures. The rating scales were the same as those used in the regulation phase.

In each phase, there were 50 trials for each experimental condition [“experimental block” (affect labeling versus affect matching versus viewing) × “emotion category” (negative versus neutral)], leading to 300 trials in Phase 1 and 300 trials in Phase 2. The complete experiment (including the preparation of the participants) lasted approximately 2 h.

### 2.4 Electroencephalograms (EEGs) recordings and preprocessing

EEG signals were recorded from 33 Ag/AgCl electrodes mounted in an EasyCap electrode system (https://www.easycap.de/) by using two 32 BrainAmp amplifiers (https://www.brainproducts.com/). The AFz and FCz electrodes were used the ground and the online reference, respectively. Vertical and horizontal electrooculogram (EOG) were recorded with additional electrodes placed above and below the right eye and outside the outer canthi of both eyes. Both the EEG and EOG data were digitized at a sampling rate of 1000 Hz, with a bandwidth between 0.016 and 100 Hz and a 50 Hz notch filter online. All electrodes had an impedance of below 10 kΩ.

Preprocessing was performed using the BrainVision Analyzer 2.0 software (https://www.brainproducts.com/). EEG data were re‐referenced to the average of the TP9 and TP10 electrodes (electrodes that correspond to mastoids) and filtered with a 30 Hz low‐pass filter (24 db/oct; zero‐phase). Ocular movements were removed from the EEG signals using the approach proposed by Gratton et al. (1983). The EEG was then segmented from −200 to 2500 ms relative to the onset of target pictures during the regulation task in Phase 1 and from −200 to 1000 ms immediately after the task in Phase 1 and during Phase 2. Trials were rejected if their voltage exceeded +100 or −100 μV at any channels. In general, 86% of trials were retained, leading to approximately 43 trials on average. The rejection rates were similar among experimental blocks during Phase 1 and between Phase 1 and 2.

Based on previous studies (Cuthbert et al., 2000; Hajcak et al., 2006; Herbert et al., 2013), LPP was measured at the Pz electrode. For the regulation phase, the effect of emotion regulation strategies on LPP responses have been suggested to occur only at specific time ranges. For example, the effect of reappraisal on the LPP response to negative stimuli starts at the time range between 1000 and 2000 ms (Paul et al., 2013; Schönfelder et al., 2014; Thiruchselvam et al., 2011). Given that the processing of affect labeling involves reappraisal, we assume that the effect of affect labeling on LPP responses might also start at the relevant time range. Accordingly, the present study separated the LPP into early and late ones at the time point of 1500 ms (the middle time point of 1000–2000 ms). That is, the LPP component was analyzed separately in these time windows, that is, early LPP (400–1500 ms) and late LPP (1500–2500 ms). Thiruchselvam et al. (2011) suggested that the lasting effect of emotion regulation did not vary with the time window of LPP. There seemed to be also the case for the current study based on Figures 4 and 6. Therefore, the LPP elicited by the target pictures immediately after the regulation task in Phase 1 and by the pictures in Phase 2 was analyzed in a time window between 400 and 1000 ms. Early and late LPPs in the regulation task of Phase 1 were calculated as mean amplitude separately from 400 to 1500 ms and from 1500 and 2500 ms, respectively, and the LPPs immediately after the regulation task in Phase 1 and in Phase 2 were measured as mean amplitude from 400 to 1000 ms.

### 2.5 Data analysis

For both Phase 1 and 2, the emotional valence and arousal ratings and the LPP amplitudes were analyzed separately by repeated‐measures analysis of variance (ANOVA), with “experimental block” (affect labeling versus affect matching versus viewing) and “emotion category” (negative versus neutral) as within‐subject factors. We also analyzed reaction accuracy and times in the regulation task by a repeated‐measures ANOVA with “experimental block” (affect labeling versus affect matching) as a within‐subject factor. For the analysis of reaction times, only trials with correct responses were included.

## 3 RESULTS

### 3.1 Behavioral data

#### 3.1.1 Phase 1

##### Valence ratings

The analysis of the valence ratings showed main effects of “experimental block” (*F*(2, 62) = 6.43, *p* = .003,${\;\eta }_{p}^{2}$ = .17) and “emotion category” (*F*(1, 31) = 282.72, *p* < .001,${\;\eta }_{p}^{2}$ = .90). The pictures were generally rated as less unpleasant in the affect labeling and viewing conditions than in the affect matching condition (*p* = .015 and .017, respectively; corrected by Bonferroni). The differences between the affect labeling and viewing conditions did not reach statistical significance (*p* = 1.000; corrected by Bonferroni). Negative pictures were rated as more unpleasant than neutral pictures.

The interaction between “experimental block” and “emotion category” was significant (*F*(2, 62) = 3.93, *p* = .025,${\;\eta }_{p}^{2}$ = .11; see Table 1). Further analyses showed that the effect of “experimental block” was significant for negative pictures (*F*(2, 62) = 6.93, *p* = .002,${\;\eta }_{p}^{2}$ = .18). These pictures were evaluated as less unpleasant in the affect labeling and viewing conditions than in the affect matching condition (*p* = .020 and .007, respectively; corrected by Bonferroni). The differences between the affect labeling and viewing conditions were not significant (*p* = 1.000; corrected by Bonferroni). For neutral pictures, there was not a significant effect of “experimental block” (*p* = .556).

**TABLE 1:** The *mean* valence and arousal ratings and their *SDs* in Phase 1 and 2.

**Phase**	**Emotion category**	**Experimental block**					
		Affect labeling		Affect matching		Viewing	
		*M*	*SD*	*M*	*SD*	*M*	*SD*
*Valence ratings*							
Phase 1	Negative	3.23	0.67	3.00	0.73	3.22	0.70
	Neutral	4.84	0.26	4.81	0.32	4.85	0.31
Phase 2	Negative	3.27	0.75	3.29	0.73	3.29	0.74
	Neutral	4.82	0.31	4.82	0.25	4.75	0.32
*Arousal ratings*							
Phase 1	Negative	5.64	1.34	5.67	1.42	5.59	1.55
	Neutral	3.93	1.56	4.03	1.48	3.91	1.46
Phase 2	Negative	5.54	1.56	5.59	1.61	5.51	1.59
	Neutral	3.85	1.50	3.81	1.50	3.92	1.50

*Note*: During Phase 1, emotional valence ratings were lower for negative pictures in the affect matching condition than in the affect labeling and viewing conditions.

##### Arousal ratings

ANOVA revealed only a main effect of “emotion category” (*F*(1, 31) = 96.09, *p* < .001,${\;\eta }_{p}^{2}$ = .76), with higher arousal for negative pictures than for neutral pictures (see Table 1).

##### Reaction accuracy and times of key presses in the affect labeling and matching tasks

The analysis did not show an effect of “experimental block” on reaction accuracy (affect labeling versus matching: 76.56% ± 8.10% versus 76.59% ± 8.13%; *p* = .987) or times (1629.42 ± 158.70 ms versus 1581.30 ± 202.12 ms; *p* = .169). The findings indicate that task difficulty was not significantly different between the affect labeling and matching tasks.

#### 3.1.2 Phase 2

##### Valence ratings

The analysis showed only a main effect of only “emotion category” (*F*(1, 31) = 189.82, *p* < .001,${\;\eta }_{p}^{2}$ = .86; Table 1). Negative pictures were evaluated as more unpleasant than neutral pictures.

##### Arousal ratings

ANOVA showed a main effect of “emotion category” (*F*(1, 31) = 96.65, *p* < .001,${\;\eta }_{p}^{2}$ = .76) but not “experimental block” (*p* = .780). The arousal ratings were higher for negative pictures than for neutral pictures. The two‐way interaction between “emotion category” and “experimental block” was significant (*F*(2, 62) = 3.64, *p* = .032, $\eta_{p}^{2}$ = .11; see Table 1). Nevertheless, further analysis did not reveal an effect of “experimental block” irrespective of “emotion category” (*p*s ≥ .072).

### 3.2 ERP data

#### 3.2.1 LPP responses during the regulation task (Phase 1)

##### Early LPP

ANOVA showed main effects of “emotion category” (*F*(1, 31) = 23.23, *p* < .001,${\;\eta }_{p}^{2}$ = .48) and “experimental block” (*F*(2, 62) = 22.32, *p* < .001,${\;\eta }_{p}^{2}$ = .42). The amplitudes were generally larger for negative pictures than for neutral pictures. Importantly, the amplitudes were larger for pictures in the affect labeling and viewing conditions than in the affect matching condition (both *p*s < .001; corrected by Bonferroni), and there were no differences between the affect labeling and viewing conditions (*p* = .722; corrected by Bonferroni).

**FIGURE 2:**
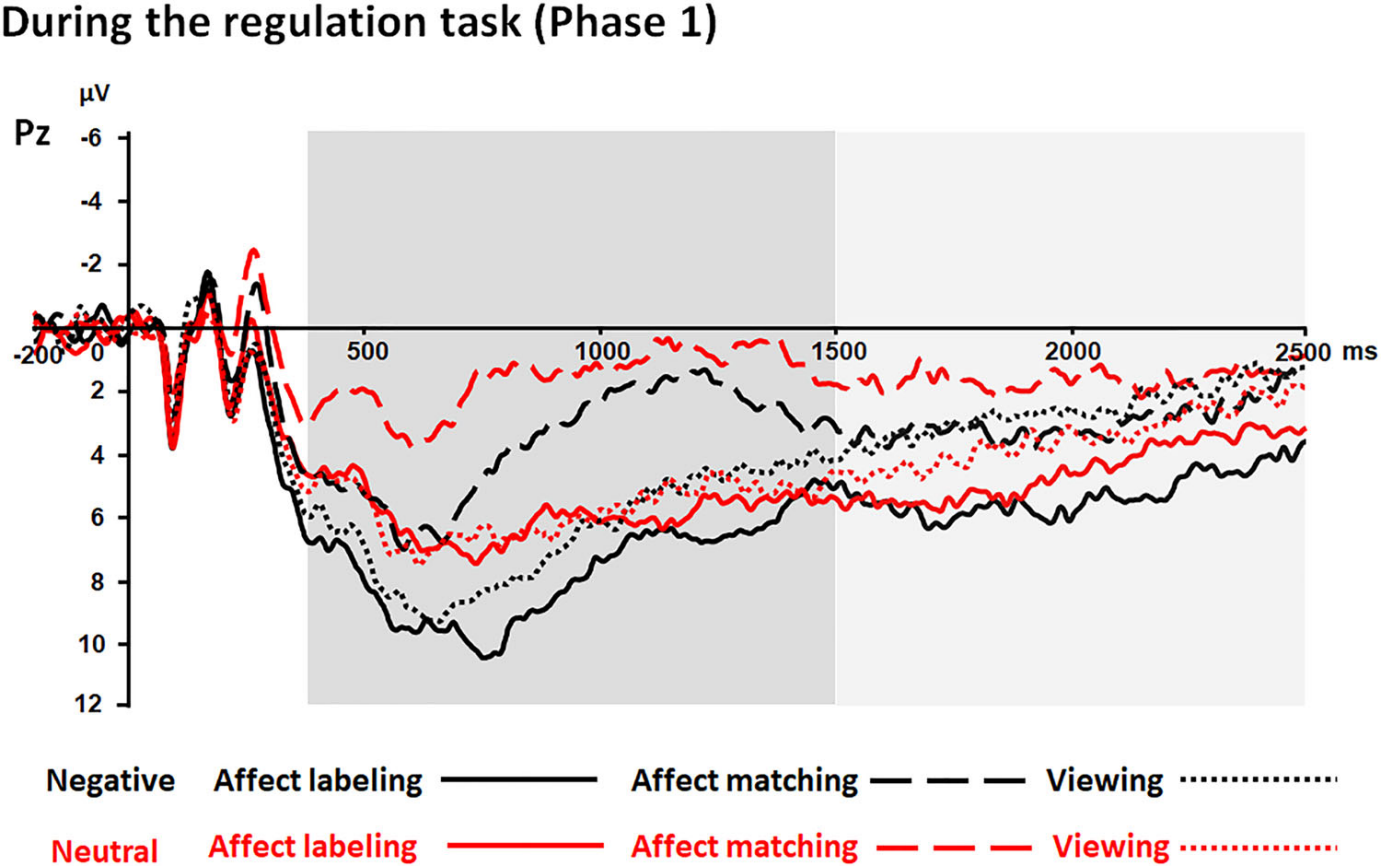
ERPs during the regulation task of Phase 1 at the Pz electrode. Shaded areas correspond to the LPP in the time windows between 400 and 1500 ms and between 1500 and 2500 ms. The early LPP amplitudes (400–1500 ms) were generally reduced in the affect matching condition irrespective of “emotion category.” This effect was sustained until the time window between 1500 and 2500 ms for neutral pictures. However, for negative pictures, there were larger responses in the affect labeling condition than in the other conditions.

**FIGURE 3:**
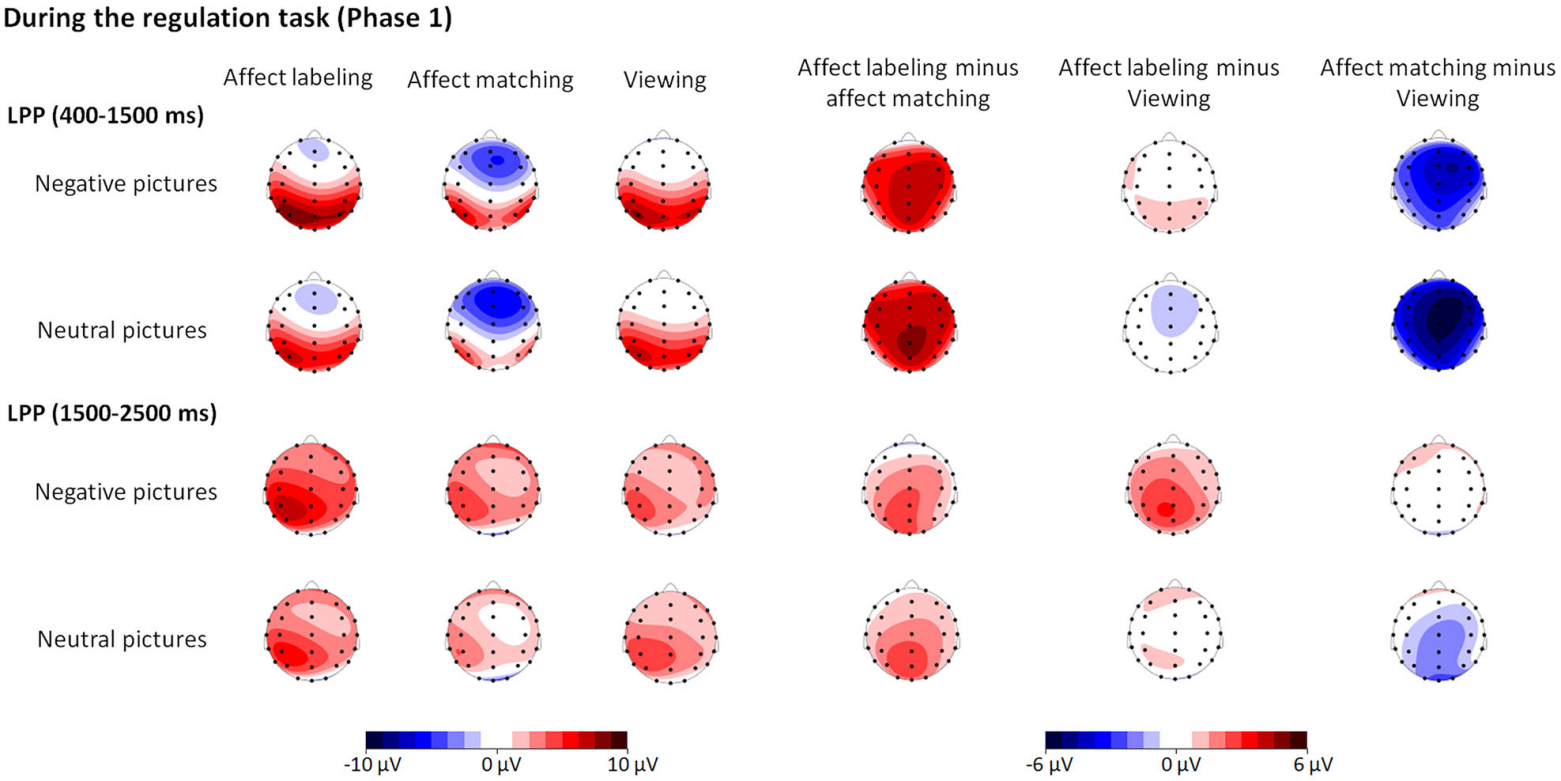
Topographical maps based on LPP mean amplitudes during the regulation task of Phase 1 and on the differences in amplitudes between each two levels of “experimental block” separately for negative and neutral pictures.

There was a trend toward an interaction between “experimental block” and “emotion category” (*F*(2, 50) = 2.81, *p* = .080,${\;\eta }_{p}^{2}$ = .08; see Table 2 and Figures 2 and 3). Nevertheless, further analysis showed that the effects of “experimental block” for both negative and neutral pictures were similar to the abovementioned main effect of “experimental block”; i.e., the amplitudes were larger in the affect labeling and viewing conditions than in the affect matching condition (both negative and neutral: *p* ≤ .001; corrected by Bonferroni), and the differences between the affect labeling and viewing conditions were not significant (negative: *p* = .153; neutral: *p* = 1.000; corrected by Bonferroni).

**TABLE 2:** The *mean* LPP amplitudes and their *SDs* for all experimental conditions.

**Phase**	**Emotion category**	**Experimental block**					
		Affect labeling		Affect matching		Viewing	
		*M*	*SD*	*M*	*SD*	*M*	*SD*
During the regulation task (Phase 1)—early LPP	Negative	7.63	5.10	3.57	6.03	6.39	5.01
	Neutral	5.91	5.23	1.30	5.80	5.76	4.82
During the regulation task (Phase 1)—late LPP	Negative	5.26	5.86	2.95	5.30	2.58	5.23
	Neutral	4.50	5.81	1.34	5.12	3.46	4.23
Immediately after the regulation task (Phase 1)—LPP	Negative	–3.14	3.77	–1.48	4.20	–1.89	3.87
	Neutral	–3.37	3.74	–3.57	3.61	–2.58	3.59
Long after the regulation task (Phase 2)—LPP	Negative	6.46	4.32	6.70	4.56	7.54	4.59
	Neutral	4.49	4.22	5.11	4.41	4.38	4.40

*Note*: During the regulation task in Phase 1, early LPPs (400–1500 ms) were larger in the affect labeling and viewing conditions than in the affect matching condition. In the late LPP (1500–2500 ms) time window, the effect of “experimental block” was sustained for neutral pictures. For negative pictures, however, late LPPs were larger than in the affect labeling condition than in the affect matching and viewing conditions. Immediately after the regulation task in Phase 1, LPP responses were smaller for negative pictures in the affect labeling condition than in the other conditions. When target pictures were presented again long after the regulation task in Phase 2, LPP responses were less positive for negative pictures in the affect labeling condition than in the viewing condition.

##### Late LPP

There was a main effect of “experimental block” (*F*(2, 62) = 7.49, *p* = .001,${\;\eta }_{p}^{2}$ = .20) but not a main effect of “emotion category” (*p* = .223). The amplitudes were generally larger in the affect labeling condition than in the affect matching condition (*p* = .002; corrected by Bonferroni), and slightly larger in the affect labeling condition than in the viewing condition (*p* = .098; corrected by Bonferroni).

The interaction between “emotion category” and “experimental block” was significant (*F*(2, 62) = 3.19, *p* = .048,${\;\eta }_{p}^{2}$ = .09; see Table 2 and Figures 2 and 3). For negative pictures, there was a significant effect of “experimental block” (*F*(2, 62) = 6.01, *p* = .004,${\;\eta }_{p}^{2}$ = .16). The amplitudes were larger in the affect labeling condition than in the affect matching and viewing conditions (*p* = .005 and .035, respectively; corrected by Bonferroni), and the differences between the affect matching and viewing conditions were not significant (*p* = 1.000; corrected by Bonferroni). There was also an effect of “experimental block” for neutral pictures (*F*(2, 62) = 6.17, *p* = .004,${\;\eta }_{p}^{2}$ = .17). The amplitudes were larger in the affect labeling and viewing conditions than in the affect matching condition (*p* = .008 and .05, respectively; corrected by Bonferroni), and the differences between the affect labeling and viewing conditions were not significant (*p* = .835; corrected by Bonferroni).

##### LPP responses immediately after the regulation task (Phase 1)

ANOVA revealed main effects of “emotion category” (*F*(1, 31) = 12.88, *p* < .001,${\;\eta }_{p}^{2}$ = .29) and “experimental block” (*F*(2, 62) = 4.63, *p* = .013,${\;\eta }_{p}^{2}$ = .13). The amplitudes were generally larger for negative pictures than for neutral pictures. The response was smaller for pictures in the affect labeling condition than in the viewing condition (*p* = .034; corrected by Bonferroni), but there was not a difference between the affect labeling and matching conditions or between affect matching and viewing conditions (both *p*s ≥ .076; corrected by Bonferroni).

**FIGURE 4:**
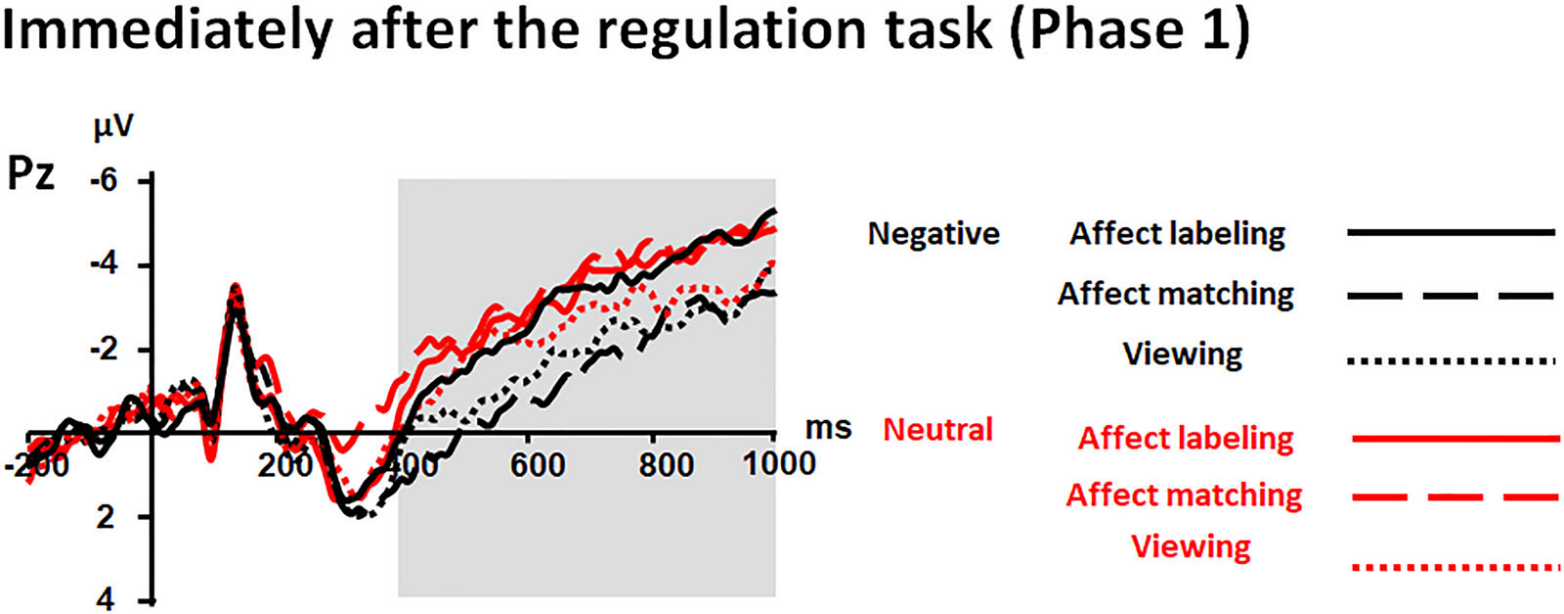
ERPs elicited by target pictures immediately after the regulation task of Phase 1 at the Pz electrode. Shaded areas correspond to the time window for LPPs (400–1000 ms). The LPP amplitudes were smaller for negative pictures in the labeling condition than in the affect matching and viewing conditions.

**FIGURE 5:**
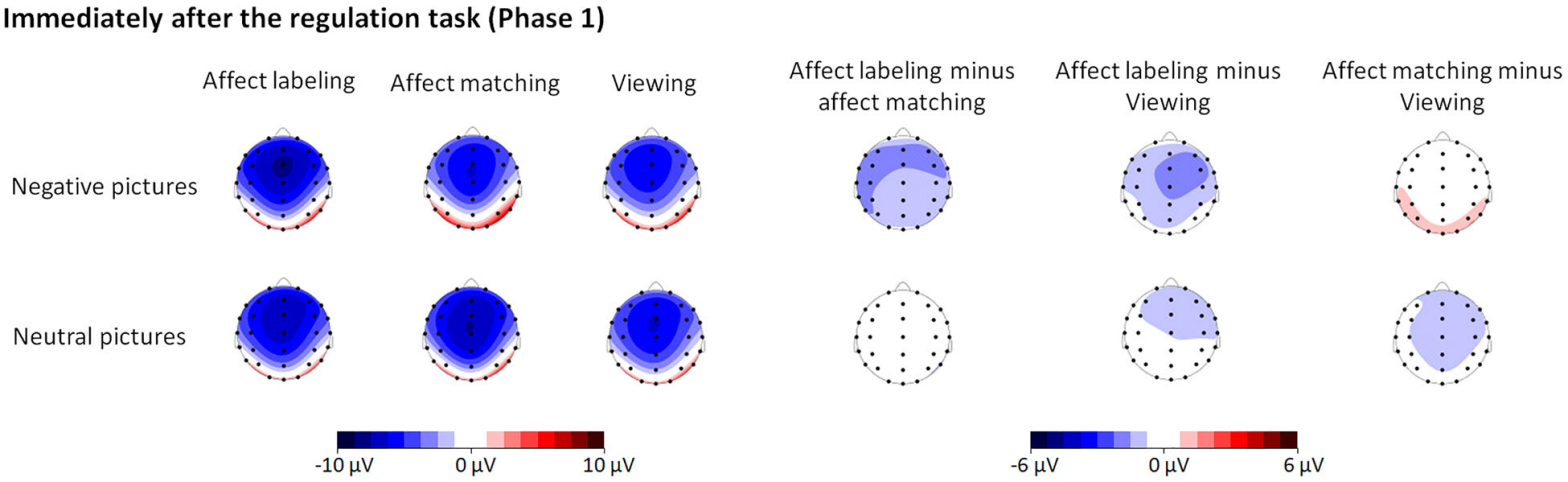
Topographical maps based on LPP mean amplitudes for target pictures immediately after the regulation task of Phase 1 and based on the differences of the amplitudes between each two levels of “experimental block” separately for negative and neutral pictures.

More importantly, the interaction between “emotion category” and “experimental block” was significant (*F*(2, 62) = 3.77, *p* = .029,${\;\eta }_{p}^{2}$ = .11; see Table 2 and Figures 4 and 5). Further analysis revealed an effect of “experimental block” on LPP responses to negative pictures (*F*(2, 62) = 7.19, *p* = .002,${\;\eta }_{p}^{2}$ = .19) but not to neutral pictures (*p* = .150). Regarding negative pictures, the LPP response was smaller in the affect labeling condition than in the affect matching and viewing conditions (*p* = .007 and .032, respectively; corrected by Bonferroni).

##### LPP responses long after the regulation task (Phase 2)

ANOVA revealed an effect of “emotion category” (*F*(1, 31) = 47.07, *p* < .001,${\;\eta }_{p}^{2}$ = .60) but not “experimental block” (*p* = .198). The amplitudes were generally larger for negative as compared to neutral pictures.

**FIGURE 6:**
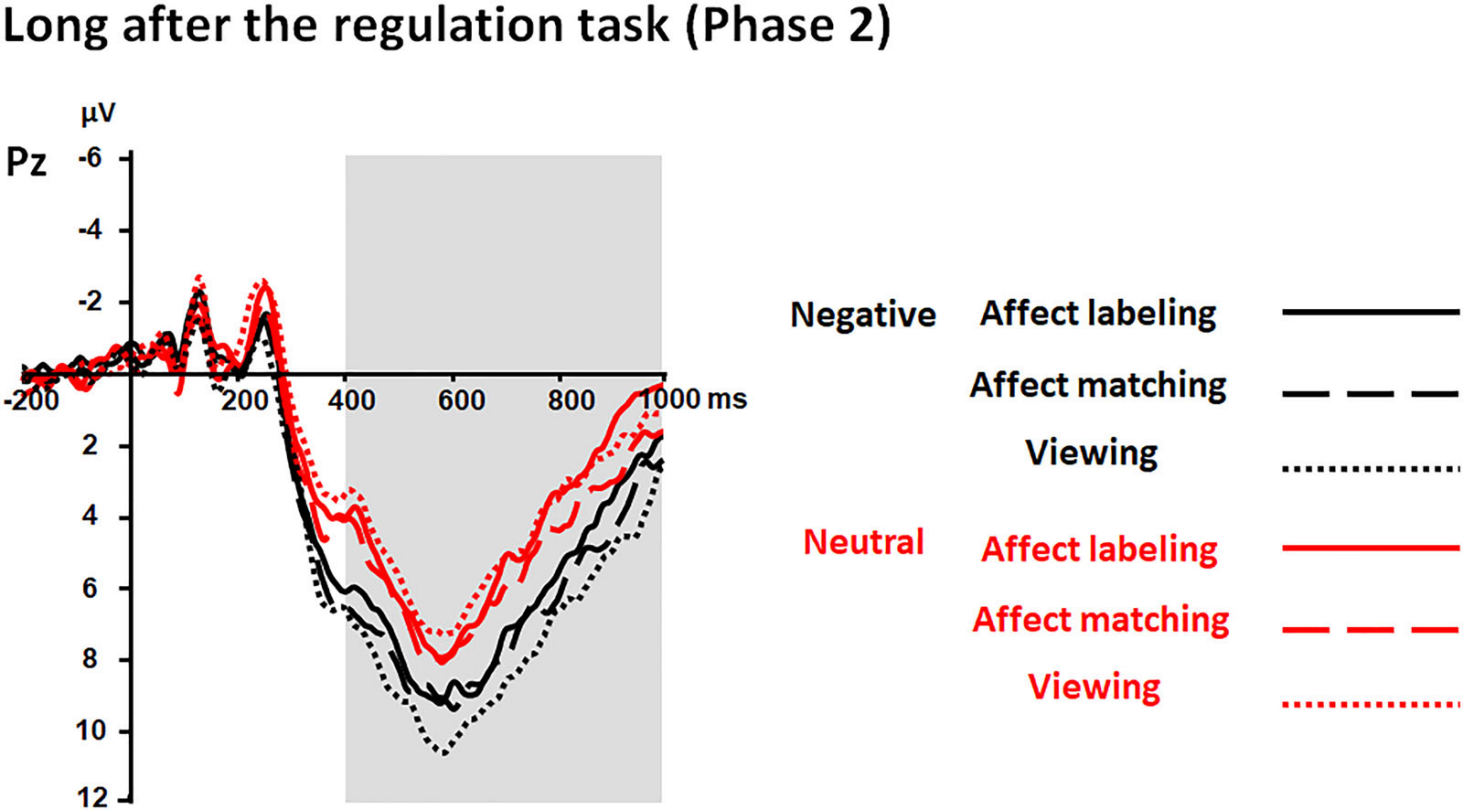
ERPs elicited by target pictures long after the regulation task in Phase 2 at the Pz electrode. Shaded areas correspond to the time window for LPPs (400–1000 ms). The LPP amplitudes were smaller for negative pictures in the labeling condition than in the viewing condition.

**FIGURE 7:**
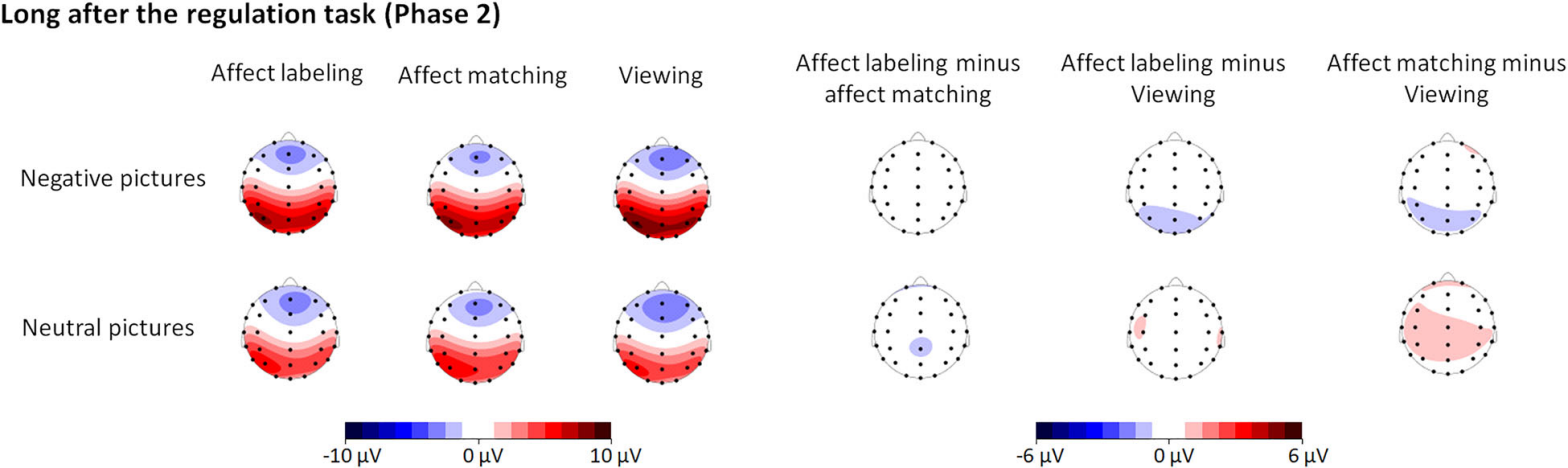
Topographical maps based on LPP mean amplitudes long after the regulation task in Phase 2 and based on the differences of the amplitudes between each two levels of “experimental block” separately for negative and neutral pictures.

Moreover, the interaction between “emotion category” and “experimental block” was significant (*F*(2, 62) = 3.25, *p* = .045,${\;\eta }_{p}^{2}$ = .10; see Table 2 and Figures 6 and 7). Further analysis revealed that the effect of “experimental block” was specific to negative pictures (*F*(2, 62) = 3.55, *p* = .035,${\;\eta }_{p}^{2}$ = .10) but not to neutral pictures (*p* = .212). With respect to negative pictures, the LPP was less positive for negative pictures in the affect labeling condition than in the viewing condition (*p* = .015), but the differences between the affect labeling and affect matching conditions and between the affect matching and viewing conditions were not significant (both *p*s ≥ .198).

## 4 DISCUSSION

We in the present study investigated both current and lasting influences of affect labeling on LPP responses. During the regulation task, results showed that LPP responses at an earlier time range (400–1500 ms) were larger in the affect labeling and viewing conditions than in the affect matching condition, irrespective of emotion category. This effect was sustained until a later time range of LPP (1500–2500 ms) for neutral pictures. However, for negative pictures, LPP (1500–2500 ms) responses were stronger in the affect labeling condition than in the affect matching and viewing conditions. The findings might suggest that affect labeling has a current influence on neural responses to negative stimuli, particularly in the late time range of LPP. When target pictures were re‐exposed soon after the regulation task, the LPP response was weaker for negative pictures in the affect labeling‐history condition than in the affect matching‐history and viewing‐history conditions. Moreover, the differential LPP responses between the affect labeling‐history and viewing‐history conditions maintained long after the regulation task. These findings might suggest that affect labeling has a lasting effect on negative stimulus processing, particularly when affect labeling is compared to mere viewing.

### 4.1 The absence of the current effect of affect labeling on early LPP responses

In the early time range of LPP (400–1500 ms), there were different LPP responses between the affect labeling and matching conditions. However, given that there were also different responses between the affect matching and viewing conditions but nonsignificant differences between the affect labeling and viewing conditions, the effect of “experimental block” might have been associated with reduced LPP responses in the affect matching condition. Reduced LPP responses might be because there was one alternative picture always to be negative in the affect matching condition, and the alternative picture might occupy attention allocated to other pictures—in particular target pictures—and thus, generally reduce attentional allocation to target pictures reflected by LPP.

### 4.2 Current effects of affect labeling on late LPP responses to negative pictures

At a later time range (1500–2500 ms), the late LPP effect for neutral pictures was not altered, whereas for negative pictures, LPP responses were stronger in the affect labeling condition than in the affect matching condition, and even in the viewing condition. The findings suggest that late LPP responses to negative stimuli are not affected by affect matching but rather by affect labeling.

Enhanced late LPP responses might indicate that affect labeling increases attention toward negative stimuli. It has been suggested that affect labeling includes ways to act upon specific emotions by activating prior conceptual knowledge of emotion categories (Barrett, 2006). This activation may involve complex cognitive processes (e.g., evaluate emotion intensity, recall past experience, etc.) and thus, may require a larger amount of attentional resources.

An alternative explanation is that affect labeling is associated with linguistic processing. It has been suggested that linguistic processes inhibit the processing of negative stimuli (Borkovec et al., 1993; Lieberman et al., 2007). The inhibitory effect on emotional responses could be reflected by LPPs (Hajcak et al., 2006; Kessel et al., 2013; Liao et al., 2019). Thus, in the present study, the effect of affect labeling on late LPPs might be associated with enhanced inhibition of negative emotional responses. This interpretation appears to be consistent with the findings of fMRI studies that showed that affect labeling increased activation in brain regions associated with inhibitory processes (e.g., the ventrolateral prefrontal cortex; Burklund et al., 2014; Lieberman et al., 2007; Memarian et al., 2017; Torrisi et al., 2013; Tupak et al., 2014).

A claim could be made that affect labeling operates through distraction, that is, requiring the performance a cognitive task to distract the participants from fully processing the emotional stimuli (Torrisi et al., 2013). However, it was found that distraction reduced LPP responses to negative stimuli (Dunning & Hajcak, 2009; Paul et al., 2013; Schönfelder et al., 2014; Thiruchselvam et al., 2011), whereas the present study found that LPP responses—particularly in the time range 1500–2500 ms—were stronger for the affect labeling condition than for the control condition. Due to different neural mechanisms, the operation of affect labeling might not—at least not fully—be explained by distraction. In addition, the operation of affect labeling could not be interpreted by attentional/cognitive load issues, because the results in the section “Reaction accuracy and times of key presses in the affect labeling and matching tasks” did not show differential response accuracy and times between the affect labeling and matching tasks, implying similar attentional/cognitive loads at least in these two conditions.

The current findings are partially consistent with the findings of Hajcak et al.’s (2006) study. In that study, LPP (500–650 ms) responses were stronger in the affect labeling condition than in the control condition, when participants in the control condition paid attention to the nonemotional aspects of the target pictures. Taking the results of Hajcak et al.’s (2006) study and the current results together, the findings indicate that there are differences in LPP effects between the affect labeling and control conditions, irrespective of whether attention is shifted to the emotional or nonemotional content of negative stimuli in the control condition. However, in Hajcak et al.’s (2006) study, viewing was not used as a control condition, making it unclear whether the LPP effect was associated with increased LPP responses in the affect labeling condition, reduced responses in the control condition or both. In the present study, the viewing condition was included as another control condition. The findings suggest that the differences in LPP responses between the affect labeling and matching conditions within a similar time range (i.e., 400–1500 ms) were associated with affect matching. The LPP effect associated with affect labeling took place within a later LPP time range (i.e., 1500–2500 ms).

Inconsistent with the findings of the present study, Herbert et al. (2013) reported smaller LPP responses in the automatic affect labeling condition (i.e., viewing labels) than in the viewing condition. Furthermore, they did not report any effects of affect labeling when the labeling was intentional (i.e., regulating the emotion of the subsequent target stimuli using the labels). In this study, the labels were presented prior to the target stimuli. Attentional processes elicited by the labels might have occurred before the onset of the target stimuli. The earlier occurrence might have influenced the LPP responses to target stimuli (Lin et al., 2012, 2015, 2018, 2020) and thus the effect of affect labeling.

In addition, our present study also found that affect labeling influenced neural responses to negative stimuli only at the time window of late LPP but not at the early LPP time window. This finding was congruent with past studies, which showed that the effect of emotion regulation strategies varied with the LPP time range during the regulation task (Schönfelder et al., 2014; Thiruchselvam et al., 2011). Similar to affect labeling, reappraisal was also found to influence neural responses only at the late LPP time window (>1000–2000 ms) but not at earlier time windows (Paul et al., 2013; Schönfelder et al., 2014; Thiruchselvam et al., 2011). Nevertheless, several other strategies (e.g., distraction) altered the LPP responses at earlier time windows but not the very late ones (e.g., >4000 ms; Thiruchselvam et al., 2011). Taken previous studies and the current study together, emotion regulation might only alter emotional reactivity in specific time ranges.

### 4.3 Lasting effects of affect labeling on LPP responses to negative pictures

When target pictures were presented immediately after the regulation task, preceding affect labeling reduced LPP responses to negative pictures to a larger extent than preceding affect matching and viewing. When target pictures were re‐exposed again long after the regulation task, LPP responses were weaker for negative pictures in the affect labeling‐history condition than in the viewing condition. The findings might suggest a lasting effect of affect labeling on the processing of negative stimuli particularly when affect labeling is compared to mere viewing. The current finding might be consistent with several previous studies, which showed that affect labeling strengthened physiological responses (e.g., skin conductance) during the regulation task but weakened the responses after the task (Kircanski et al., 2012; Mendolia & Kleck, 1993; Niles et al., 2015; Tabibn et al., 2008).

A model of affective adaptation proposed by Wilson and Gilbert (2008) proposes that when individuals encounter emotional events, they increase attention toward the events and attempt to explain or appraise events. If individuals succeed, they will attend less to the events and have weaker affective reactions to the events. Accordingly, for our present study, affect labeling may increase attention to activates conceptual knowledge of emotion categories, which involves emotional evaluations, comparisons with past experience, etc. (Barrett, 2006). These processes might be helpful for reappraising negative stimuli (Lieberman et al., 2011) and reduce attention toward negative stimuli after successful reappraisal (Paul et al., 2013; Schönfelder et al., 2014; Thiruchselvam et al., 2011). The reduced attention might lead to reduced LPP responses toward relevant stimuli when they are presented again.

An alternative explanation is that affect labeling inhibits the processing of negative stimuli (Borkovec et al., 1993; Lieberman et al., 2007). Successful inhibition might be helpful in reducing emotional responses to negative stimuli. This reduced response might be sustained when the individuals are re‐presented to the negative stimuli immediately and even long after the regulation task, leading to the reduction of LPP responses.

However, there were no differences between the affect labeling and matching conditions long after the regulation task. Therefore, the effect of affect labeling on negative stimulus processing could be maintained for long only when affect labeling is compared to specific control conditions (e.g., viewing) but not to other conditions (e.g., affect matching).

Moreover, as mentioned above, alternative negative pictures in each trial might occupy attention resources allocated to target stimuli and thus, decrease emotional responses. Nevertheless, when negative pictures without any alternative pictures were presented again soon after the regulation task, the LPP response was larger in the affect matching condition, at least compared to the affect labeling condition, suggesting that attention is maintained to further process negative target pictures to some extent. This maintained attention might decrease emotional reactivity when negative target stimuli are subsequently presented for the third time long after the regulation task (Wilson & Gilbert, 2008), resulting in reduced the differences in LPP responses between the affect labeling and matching conditions. Nevertheless, the further process might be weak because there were no differences between the affect matching and viewing conditions soon and long after the regulation task.

### 4.4 The absence of current and lasting effects of affect labeling on subjective experience

The present study did not reveal an influence of affect labeling on valence or arousal ratings, irrespective of the emotional category and experimental phase. Affect labeling is thought to be an implicit regulatory strategy that occurs without insight or awareness (Gyurak et al., 2011; Koole & Rothermund, 2011; Koole et al., 2015). The insignificant effect of affect labeling in the present study might have occurred because this unconscious regulation could not be reflected by conscious measures (e.g., subjective experiences; e.g., Constantinou et al., 2014; Herbert et al., 2013; Niles et al., 2015). This assumption is consistent with the study by Lieberman et al. (2011), in which individuals did not consider affect labeling was an effective regulatory strategy, even though affect labeling actually led to lower emotional responses.

### 4.5 Limitations and outlines for future studies

Finally, we would like to mention some of the limitations of the current study and make suggestions for future studies. First, the current study did not find an effect between the affect labeling and matching conditions on LPP responses to negative stimuli long after the regulation task. As mentioned above, the insignificant effect might be associated with the re‐exposure of emotional stimuli immediately after the regulation task. Future studies might not present the stimuli immediately after the task and further investigate whether there will be a long lasting effect between the affect labeling and matching conditions. Second, for the current study, the labels were limited to “neutral” and “negative.” Future studies might use more specific labels (e.g., angry and fearful) to further investigate the related issues. Third, participants were required to rate the emotional valence and arousal degree of the pictures to improve our understanding of the behavioral effects of affect labeling. However, some studies have suggested that emotional ratings serve as an implicit regulation strategy that might influence the processing of emotional pictures (Hutcherson et al., 2005; Taylor et al., 2003). Future studies may avoid asking participants to rate the pictures to gain further understanding of the effects of affect labeling. Finally, future studies might investigate whether there are any other lasting effects of affect labeling, such as long‐term memory effects.

## 5 CONCLUSION

The present study found that affect labeling immediately resulted in larger neural responses to negative pictures in the late time range of LPP than did affect matching and viewing. When the pictures were presented immediately after the affect labeling, affect matching and viewing tasks, LPP responses were smaller for negative pictures with a history of affect labeling than those with a history of affect matching and viewing. Moreover, the difference between the affect labeling and viewing conditions was maintained long after the regulation task. Taken together, the current findings indicate that negative stimulus processing is upregulated when affect labeling is used but to some extent downregulated after affect labeling is completed.

## CONFLICT OF INTEREST STATEMENT

The authors declare no competing interests.

### ETHICS STATEMENT

The study was approved by the Academic Committee of School of Public Administration, Guangdong University of Finance.

### PATIENT CONSENT STATEMENT

All participants provided written informed consent in line with the standard ethical guidelines of the Declaration of Helsinki.

### PERMISSION TO REPRODUCE MATERIAL FROM OTHER SOURCES

No materials are reproduced from other sources for the current study.

### CLINICAL TRIAL REGISTRATION

Our present study does not involve clinical trials.

### PEER REVIEW

The peer review history for this article is available at https://publons.com/publon/10.1002/brb3.3065

## Data Availability

The data that support the findings of this study are available from the corresponding author upon reasonable request.
